# Correlation between follicular fluid hormonal levels in PCOS women and embryo development in ART cycles

**DOI:** 10.1371/journal.pone.0342463

**Published:** 2026-02-09

**Authors:** Sunatchana Kongsomnuan, Pornsri Niransuk, Artitaya Singwongsa, Chonthicha Satirapod

**Affiliations:** Department of Obstetrics and Gynaecology, Reproductive Endocrinology and Infertility Unit, Ramathibodi Hospital, Mahidol University, Bangkok, Thailand; Bowen University, NIGERIA

## Abstract

Polycystic ovarian syndrome (PCOS) is a common endocrine disorder characterized by ovulatory dysfunction. Fertility outcomes in PCOS patients are often suboptimal, potentially owing to alterations in the follicular fluid (FF) microenvironment. However, the differences in FF hormone levels between PCOS and non-PCOS patients, as well as their correlation with assisted reproductive technology (ART) outcomes, remain unclear. This prospective study included 18 PCOS patients and 18 infertile women without PCOS (control group) undergoing intracytoplasmic sperm injection at the Division of Reproductive Medicine, Ramathibodi Hospital. The primary objective was to compare ART outcomes between the groups. Furthermore, FF testosterone, dehydroepiandrosterone sulfate, and luteinizing hormone levels were evaluated to assess their correlation with these outcomes. The number of retrieved oocytes was significantly higher in the PCOS group; however, the rates of metaphase II oocyte formation, fertilization, blastocyst formation, and high-quality blastocyst formation were comparable between the groups. Although FF testosterone and FF luteinizing hormone levels were higher in the PCOS group than in the control group, the differences were not statistically significant. Spearman correlation analysis showed that FF testosterone levels were negatively correlated with fertilization rate (r = −0.3496, p = 0.0366). These findings suggest that increased FF testosterone levels may negatively correlation with fertilization rates, which may reflect one of the contributing factors to the suboptimal ART outcomes observed in PCOS patients.

## Introduction

Polycystic ovarian syndrome (PCOS) is a prevalent reproductive disorder in females, affecting approximately 8%–13% of women of reproductive age [1]. Its clinical manifestations include menstrual irregularities, hyperandrogenic symptoms, and, in many cases, obesity [2]. Diagnosis is supported by transvaginal ultrasound, typically revealing polycystic ovarian morphology. The pathophysiology of PCOS is multifactorial, involving hyperandrogenism, insulin resistance, anovulation, and genetic predisposition [3]. Although PCOS primarily affects young women, those with anovulation often experience infertility. In such cases, assisted reproductive technologies, including in vitro fertilization (IVF) or intracytoplasmic sperm injection (ICSI), may be considered when ovulation induction fails [4].

Women with PCOS who exhibit polycystic ovarian morphology typically produce a higher number of oocytes during ovarian stimulation than those without PCOS [5–7]. While some studies report comparable pregnancy and live birth rates between PCOS and non-PCOS patients [5], others indicate poorer outcomes in women with PCOS [6,7]. Oocyte quality is influenced by the follicular fluid (FF) microenvironment, which contains key factors essential for oocyte development [6]. Several studies have investigated the relationship between FF composition and IVF outcomes, particularly in PCOS patients [5–9]. Evidence suggests that FF in women with PCOS differs in its levels of steroid hormones, including androgens (testosterone, dehydroepiandrosterone sulfate), estrogens (estradiol, estrone), and progesterone [10]. Yu et al. reported a negative correlation among estriol, estradiol, and pregnenolone levels and oocyte quality in PCOS patients [6]. However, no studies have examined the correlation between androgen levels and IVF outcomes in PCOS patients. In a retrospective study of women without PCOS, Natachandra et al. observed that elevated dehydroepiandrosterone sulfate (DHEA-s) levels (>1850 ng/ml) in FF were associated with higher fertilization, cleavage, blastocyst formation, and live birth rates compared to lower DHEA-s levels [11].

Owing to abnormal gonadotropin-releasing hormone (GnRH) pulsatility, women with PCOS often exhibit elevated luteinizing hormone (LH) levels compared to those without PCOS. Yuan Wang et al. reported significantly higher FF LH levels in the PCOS group than in the control group; however, they did not investigate the correlation between FF LH levels and IVF outcomes [12]. Although some studies have supported the role of LH in folliculogenesis during ovarian stimulation in PCOS, data on LH concentrations in FF and their relationship with IVF outcomes remain limited.

Given this background, this prospective cohort study primarily aims to compare IVF outcomes between women with and without PCOS undergoing ICSI. Additionally, it examines differences in testosterone, DHEA-S, and LH levels and their correlations with IVF outcomes.

## Materials and methods

This prospective cohort study was conducted between 26 January and 11 December 2024 at the Division of Reproductive Medicine, Department of Obstetrics and Gynecology, Faculty of Medicine, Ramathibodi Hospital, Mahidol University. The Human Research Ethics Committee of the Faculty of Medicine, Ramathibodi Hospital, approved the study (protocol No. MURA2023/917, date: December 7, 2023). Written informed consent was obtained from all participants before enrollment.

The study included infertile women under 40 years of age who underwent ICSI at the Division of Reproductive Medicine. Their partners had at least one abnormal sperm parameter (e.g., teratozoospermia), necessitating ICSI. All women in the study group were diagnosed with PCOS according to the 2003 Rotterdam criteria, which require at least two of the following three features after excluding related disorders: (1) oligo- or anovulation, (2) clinical and/or biochemical signs of hyperandrogenism, and (3) polycystic ovarian morphology [13]. Patients were excluded if they had ovarian cysts, had their IVF cycles canceled, or required surgically retrieved sperm. The control group comprised women without PCOS who had regular menstrual cycles but were diagnosed with infertility due to other conditions.

### Baseline characteristics

Collected demographic data included age, underlying conditions, causes and duration of infertility, history of abortion, previous infertility treatments, and menstrual patterns. Weight and height were measured to calculate body mass index (BMI, kg/m^2^). Symptoms and signs of hyperandrogenism, including acne, oily skin, and hirsutism, were documented during physical examinations. Transvaginal ultrasound was performed to assess ovarian pathology and polycystic ovarian morphology. Male partners were interviewed regarding their medical history and underwent semen analysis. Abnormal semen parameters, including teratozoospermia, astheno-teratozoospermia, and oligo-asthenozoospermia, were identified to evaluate the severity of male factor infertility [14].

### Controlled ovarian hyperstimulation

All patients received gonadotropin-based ovarian stimulation, initiated on days 2–3 of the menstrual cycle over the planned course for oocyte pick-up, following a transvaginal ultrasound assessment of antral follicle count. Stimulation was performed using recombinant follicle-stimulating hormone or human menopausal gonadotropin according to the antagonist protocol. The gonadotropin dose was determined by a physician based on patient age, prior cycle history, and ovarian reserve. Follicular growth and count were monitored via transvaginal ultrasound, and the gonadotropin dose was adjusted if follicular development was insufficient. A GnRH antagonist was administered when at least one follicle reached 14 mm, with dosage and duration determined at the physician’s discretion. Oocyte maturation was triggered using recombinant human chorionic gonadotropin, a GnRH agonist, or both, when at least one follicle reached 18 mm. Oocyte retrieval was performed 36 h later under transvaginal ultrasound guidance using a single-lumen catheter.

### FF collection

FF was individually aspirated from ovarian follicles with a diameter of 14 mm or greater and collected into sterile tubes. A single-lumen catheter was used to prevent contamination with transfer media. After oocyte retrieval and removal, the remaining FF from all aspirated follicles in each ovary was pooled without adjusting for equal volumes per follicle, resulting in one combined sample per patient. The pooled FF was then transferred to the laboratory for hormonal analysis, including measurements of testosterone, DHEA-S, LH, and estradiol levels. To separate red blood cells and other cellular debris, the FF was centrifuged at 3,500 rpm for 10 min. Hormonal levels were analyzed using the chemiluminescence microparticle immunoassay technique (Abbott, Germany).

### Oocyte and embryo assessment

All oocytes were denuded and assessed for maturation after separation from the FF. Oocytes at the metaphase II (MII) stage were fertilized via ICSI by an experienced embryologist. The MII rate was calculated as the number of MII-stage oocytes divided by the total number of retrieved oocytes. Normal fertilization was defined as the presence of two pronuclei (2PN) observed 18–19 h after ICSI. The fertilization rate was calculated as the number of 2PN embryos divided by the total number of retrieved oocytes. Embryos were evaluated on Days 3 and 5 post-fertilization using the Gardner embryo grading system. The blastocyst development rate was defined as the number of blastocysts divided by the number of 2PN embryos. A high-quality embryo was defined as one graded 3BB or higher. The high-quality blastocyst rate was calculated as the number of high-quality embryos divided by the number of 2PN embryos [15]. To minimize bias, embryologists were blinded to the hormonal assay results, while laboratory technicians conducting hormone analyses were blinded to the outcomes of embryo development.

### Statistical analysis

The sample size was calculated for the comparison of two independent means. Yu et al. [6] reported a rate of embryos develop to blastocyst in both PCOS and control groups. In this study, the percentage different between the groups was set to 8%, resulting in a sample size of 18 patients per group. Continuous data were expressed as means with standard deviations, while categorical data were presented as frequency and percentages. The baseline characteristics of patients, IVF outcomes, and FF hormonal level were compared between PCOS and control groups using Pearson’s χ^2^ test for categorical variables and the t-test for continuous variables with normally distributed or the Mann‒Whitney U test for non-normally distributed variables. Spearman correlation analysis was used to assess relationships between hormonal levels (LH, testosterone, DHEA-S) and oocyte/embryo outcomes. Linear regression analysis was performed to identify factors potentially associated with blastocyst formation rate. Variables deemed clinically relevant were considered in the multivariate linear regression model. Statistical significance was defined at a P-value < 0.05. All statistical analyses were performed using Stata version 18.0.

## Results

### Baseline characteristics and controlled ovarian hyperstimulation outcomes

A total of 36 women were included in the study and divided into two groups: PCOS (N = 18) and control (N = 18). The baseline characteristics between PCOS and control group are presented in Table 1. Age, type of infertility, history of abortion, previous assisted reproductive technology (ART) treatment, and clinical dysmenorrhea were comparable between the groups. All women in the control group experienced regular menstrual cycles. Women with PCOS had a significantly higher BMI than those in the control group. Additionally, clinical hyperandrogenism and polycystic ovarian morphology were significantly more prevalent in the PCOS group. Regarding controlled ovarian hyperstimulation outcomes (Table 2), the stimulation duration was similar between groups. However, the control group required a significantly higher gonadotropin dose than the PCOS group. Despite a significantly higher number of retrieved oocytes in the PCOS group, the rates of MII oocytes, fertilization, blastocyst formation, and high-quality blastocyst formation were comparable between the groups.

**Table 1:** Comparison of baseline participant characteristics.

	Control (N = 18)	PCOS (N = 18)	P-value
**Age (years)**	35.89 ± 2.17	34.28 ± 3.40	0.10
**BMI (kg/ml²)**	21.23 ± 0.63	24.59 ± 1.49	0.04*
**Underlying disease (%)**	5.56%	22.22%	0.34
**Infertility duration (years)**	3.78 ± 3.36	3.86 ± 3.24	0.37
**Infertility (%)**			1.00
**- Primary**	88.89%	94.44%	
**- Secondary**	11.11%	5.56%	
**Abortion history (%)**	16.67%	44.44%	0.07
**Regular menstruation (%)**	100%	38.89%	<0.01**
**Dysmenorrhea (%)**	27.78%	33.33%	0.717
**Clinical hyperandrogenism (%)**			<0.01**
**- Acne and oily skin**	0	55.56%	
**- Hirsutism**	0	38.89%	
**PCOM (%)**	5.56%	88.89%	<0.01**
**Previous ICSI (%)**	22.22%	16.67%	1.0
**Male factor infertility (%)**			1.0
**- Teratozoospermia**	83.33%	88.89%	
**- Asteno-teratozoospermia**	5.56%	11.11%	
**- Oligo-teratozoospermia**	5.56%	0	
**- Oligoasthenoteratozoospermia**	5.56%	0	

BMI: Body mass index, PCOM: Polycystic ovarian morphology, ICSI: Intracytoplasmic sperm injection, PCOS: Polycystic ovary syndrome.

*P-value was calculated using t-test

**P-value was calculated using Pearson’s χ²

**Table 2:** Comparison of IVF outcomes and FF hormone levels.

	Control (N = 18)	PCOS (N = 18)	P-value
**Antral follicle count (*n*)**	10.5 ± 0.837	18.05 ± 1.248	<0.01*
**Gonadotropin dose (IU)**	2206.944 ± 153.43	1841.667 ± 123.21	0.07
**Stimulation duration (days)**	9.27 ± 0.240	9.33 ± 0.256	0.87
**Oocyte trigger method (%)**			
**- GnRH Agonist**	33.33%	83.33%	<0.01**
**- Dual trigger**	38.89%	16.67%	
**- hCG**	27.78%	0	
**Number of oocytes retrieved (*n*), median (IQR)**	10 (4,28)	21 (4,40)	<0.01***
**MII oocyte rate (%)**	80.70 ± 2.56	77.13 ± 4.57	0.50
**Fertilization rate (%)**	70.92 ± 4.40	71.95 ± 4.25	0.86
**Blastocyst formation rate (%)**	63.04 ± 5.16	65.79 ± 5.89	0.72
**High-quality blastocyst formation rate (%), median (IQR)**	25.0 (14.28,75)	39.8 (20,75)	0.31
**FF DHEA-S (µg/dL)**	120.89 ± 12.03	128.33 ± 13.95	0.68
**FF testosterone (ng/dL), median (IQR)**	546.75 (125.3,1862)	956.35 (183.5,3099)	0.09
**FF LH (mIU/mL), median (IQR)**	3.08 (0.1,8.48)	3.8 (0.87,15.25)	0.07

GnRH: Gonadotropin-releasing hormone, hCG: Human chorionic gonadotropin, MII: Metaphase II, FF: Follicular fluid, DHEA-S: Dehydroepiandrosterone sulfate, LH: Luteinizing hormone, PCOS: Polycystic ovary syndrome.

* P-value was calculated using t-test

** P-value was calculated using Pearson’s χ²

*** P-value was calculated using Mann–Whitney U test

### Correlation between FF profile, fertilization rate, and embryo development

FF hormonal profiles of the participants were illustrated in Table 2. FF DHEA-S levels were comparable between the groups. The PCOS group exhibited higher median FF testosterone levels (956.35 ng/dL, range: 183.5–3,099 ng/dL) than the control group (546.75 ng/dL, range: 125.3–1,862 ng/dL); however, this difference was not statistically significant (p = 0.0965). FF LH levels were also slightly higher in the PCOS group than in the control group, but the difference was not significant (3.80 vs. 3.09 mIU/mL, p = 0.0790).

**Fig 1:**
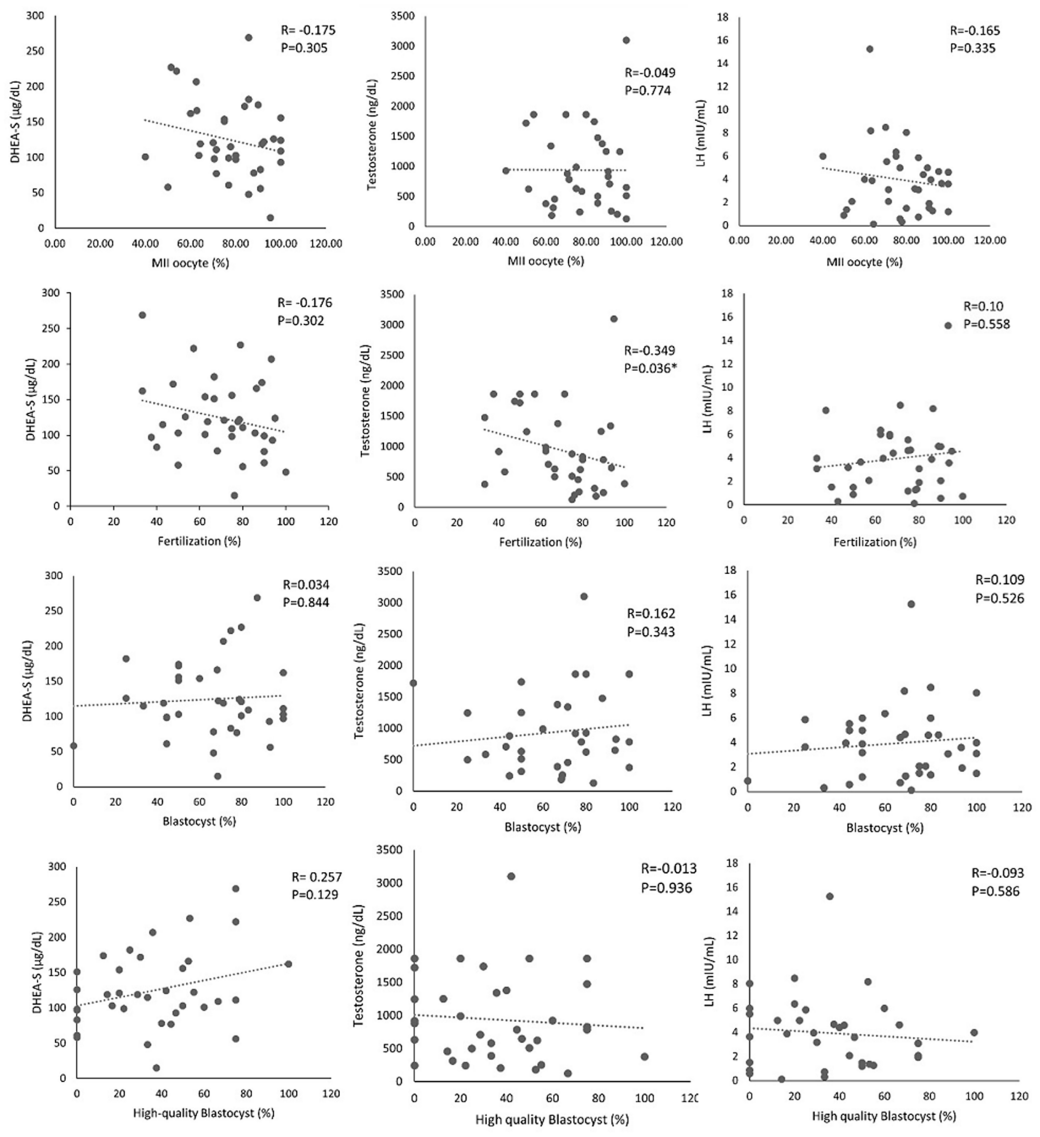
Correlation of FF DHEA-S, testosterone, and LH levels with embryo outcomes, shown in Columns 1, 2, and 3, respectively.

Although estradiol levels were assessed, all values exceeded the upper detection limit of 30,000 pg/mL, thereby precluding further quantitative analysis. This methodological limitation may have affected the completeness of the hormonal profile comparison and restricted the ability to fully explore the role of estradiol in ART outcomes. Future research should consider employing alternative assay techniques or instruments with higher analytical sensitivity to facilitate more comprehensive hormonal evaluation. Given the differences in FF hormone levels between the two groups, we aimed to determine whether these hormones could predict controlled ovarian stimulation and embryo development outcomes. Our findings revealed that elevated FF testosterone levels were significantly associated with lower fertilization rates (r = −0.3496, p = 0.0366), as shown in Fig 1. However, no significant correlation was observed between FF DHEA-S or FF LH levels and IVF outcomes.

To further investigate whether factors other than follicular fluid (FF) hormones may influence IVF outcomes, we used linear regression analysis to examine the association between various factors and blastocyst formation rate, which represents the final outcome of embryo development. First, univariate linear regression analysis was performed, none of the factors were found to have a significant impact on blastocyst formation rate, as presented in S1 Table. To complement these findings and provide a more comprehensive assessment, we also constructed multivariate linear regression models to evaluate potential predictors of fertilization and blastocyst rates. All models were adjusted for the potential confounding effects of patient age and BMI. For the outcome blastocyst rate, FF testosterone, DHEA-S, and LH remained non-significant predictors of blastocyst rate (coefficient = 0.005, 0.041, and 0.833, and P-value = 0.524, 0.649 and 0.600, respectively), as shown in S2 Table. However, when focused on fertilization rate, FF testosterone emerges as a significant independent predictor. Higher testosterone levels were significantly associated with a lower fertilization rate (coefficient = −0.011, P-value = 0.040). The other predictors, FF DHEA-S and LH, showed nonsignificant predictors (Coefficient = −0.105 and 0.900, and P-value = 0.105 and 0.427, respectively), as present in S3 Table.

## Discussion

Previous studies have typically reported poorer ART outcomes in patients with PCOS than in those without PCOS [6,7]. This observation has led to extensive research into potential causes, with a primary hypothesis that hormonal imbalances may alter the FF composition, which serves as the microenvironment for oocytes [7]. FF plays a crucial role in oocyte development, and abnormalities in its composition may negatively impact oocyte quality [16]. Bongrani et al. identified differences in FF composition between PCOS patients and controls, reporting higher FF androgen levels in the PCOS group and elevated 17OH-progesterone, deoxycorticosterone, and 11-deoxycortisol levels in the control group [10].

The primary aim of this study was to compare IVF outcomes between the PCOS and control groups. Our findings indicate that the PCOS group had a significantly higher number of retrieved oocytes, likely attributed to the greater antral follicle counts observed in individuals with polycystic ovarian morphology. Despite the higher retrieved oocyte yield in the PCOS group, the MII oocyte fertilization rate was comparable between the groups, suggesting the involvement of additional factors influencing oocyte quality [17–21]. This finding is consistent with the study by Swanton A et al., which also reported similar fertilization rates between PCOS and non-PCOS patients despite differences in the number of retrieved oocytes [7]. This may be explained by alterations in the FF microenvironment in PCOS patients consistent with the findings reported by Bongrani et al. [10]. However, further research is needed to elucidate the underlying mechanisms.

To investigate the factors contributing to poor oocyte quality, we evaluated FF hormone levels in both groups. Our analysis revealed that FF testosterone levels were higher in the PCOS group; however, the difference was not statistically significant, which may be attributable to the small sample size limiting the statistical power to detect group differences. This finding deviates from previous studies that reported a significant difference in FF testosterone levels between the PCOS and control groups [10]. Ovarian androgens, including DHEA, Δ4-androstenedione, and testosterone, are elevated in women with PCOS due to the predominant activity of CYP17A1 on 3βHSD [10]. This mechanism aligns with findings from another study, which suggested that hyperandrogenism results from increased 17-hydroxylase/17,20-lyase activity of cytochrome CYP17 in ovarian theca cells [22]. Androgen excess appears to promote the transition of follicles from the primordial to the primary stage, increasing the number of small follicles in PCOS patients and leading to polycystic ovarian morphology [17]. Another notable finding in our study was the slightly higher FF LH levels in PCOS patients compared to the control. This is consistent with previous research reporting significantly higher FF LH levels in the PCOS group than in the non-PCOS group [12]. The underlying mechanism involves elevated serum LH levels in PCOS, driven by abnormal GnRH pulsatility, which contributes to ovulatory dysfunction [17]. High serum LH stimulates excessive testosterone production by hypertrophied theca cells, leading to hyperandrogenism [23].

Many studies have examined whether FF components are associated with IVF outcomes and can serve as markers for predicting ovarian stimulation results. For instance, Yu et al. demonstrated that elevated FF estriol levels were significantly correlated with fewer MII oocytes. They also reported a significant negative correlation between FF pregnenolone levels and the blastocyst formation rate [6]. Our study revealed that increased FF testosterone levels were significantly associated with lower fertilization rates, supporting the hypothesis that androgen excess may impair oocyte quality, although androgens play a vital role in normal ovarian physiology by binding to receptors in granulosa and theca cells and enhancing follicle-stimulating hormone receptor expression [24]. Regression analysis confirmed negative correlation, higher FF testosterone predicted a lower fertilization rate. By comparison, no independent predictive factors were identified for the blastocyst formation rate. However, these findings should be interpreted with caution, as the significance was observed in the context of a relatively small sample size. Further studies with larger sample sizes are warranted to confirm these findings. If such a negative correlation is confirmed, strategies to reduce FF testosterone levels prior to ovarian stimulation could be considered as a therapeutic approach to improve IVF outcomes, including fertilization and clinical pregnancy rate.

A key strength of this study is its prospective design. While few studies have examined FF testosterone and FF LH levels in PCOS patients, this is the first attempt to investigate their correlation with IVF outcomes. The primary limitation of this study is the lack of serum sex hormone measurements, which could have provided a more comprehensive comparison with FF hormone levels. The inclusion of serum hormone data could have provided valuable insights into whether the hormonal alterations observed were reflective of systemic differences or localized changes within the ovarian microenvironment. Nevertheless, existing literature shows inconsistent correlations between serum and FF hormone levels, with some studies reporting associations that appear to be limited to specific hormones [6,10]. CMIA is an enhanced immunoassay technique for measuring hormones in biological specimens, offering exceptional sensitivity and specificity, which are essential for precise diagnosis and monitoring. However, it may be affected by structurally similar compounds. Additionally, pre-analytical factors such as hemolysis or sample contamination can disrupt hormone detection. Lastly, a limitation of this study is the small sample size. The absence of statistical significance may be attributable to this limitation and may hinder the detection of true differences between groups. In summary, given the limited sample size, the modest level of statistical significance, and the assay constraints, the results should be interpreted with caution.

## Conclusion

The results of this study revealed elevated FF testosterone and LH levels in PCOS patients, supporting the hypothesis of ovarian hyperandrogenism in PCOS. Despite the higher number of retrieved oocytes in PCOS patients, controlled ovarian stimulation outcomes were comparable to those in the control group. This finding may be attributed to increased FF testosterone levels, as our study identified a significant negative correlation between FF testosterone and fertilization rates. A larger prospective study is needed to validate these findings, which could contribute to refining infertility treatment strategies for PCOS patients. Optimizing the FF microenvironment may improve treatment outcomes in this population.

## Supporting information

S1 Table Univariate analysis of factors associated with blastocyst formation rate. (DOCX)

S2 Table Multivariate analysis of factors associated with blastocyst formation rate. (DOCX)

S3 Table Multivariate analysis of factors associated with fertilization rate. (DOCX)

S1 Dataset Primary data set of follicular fluid (FF) hormones and embryo development outcomes. (XLSX)
