# Supplementary material for: Correlation between follicular fluid hormonal levels in PCOS women and embryo development in ART cycles
Source: PLoS One. 2026 Feb 9;21(2):e0342463. doi: 10.1371/journal.pone.0342463 (PMC12885263; doi:10.1371/journal.pone.0342463)
Supplement: S1 Table — (DOCX) [file pone.0342463.s001.docx]

**S1 Table. Univariate analysis of factors associated with blastocyst formation rate**

|  | Coefficient | 95% Confident interval (CI) | P-value |
| --- | --- | --- | --- |
| **Age (years)** | -0.47 | (-3.32, 2.38) | 0.739 |
| **BMI (kg/m²)** | -0.59 | (-2.23, 1.04) | 0.468 |
| **History abortion** | 0.26 | (-17.59, 18.12) | 0.976 |
| **Regular menstruation** | -1.48 | (-19.33, 16.37) | 0.867 |
| **Infertility duration (years)** | -1.27 | (-3.72, 1.16) | 0.296 |
| **Dysmenorrhea** | 3.07 | (-14.34, 20.49) | 0.722 |
| **Hirsutism** | 6.76 | (-13.88, 27.42) | 0.510 |
| **Acne** | 2.28 | (-16.06, 20.63) | 0.802 |
| **Oily skin** | -0.80 | (-19.17, 17.56) | 0.929 |
| **PCOM** | -0.27 | (-16.75, 16.20) | 0.973 |
| **AFC (*n*)** | -0.12 | (-1.53, 1.27) | 0.853 |
| **Gonadotropin dose (IU)** | -0.001 | (-0.01, 0.012) | 0.974 |
| **Stimulation duration (days)** | -0.67 | (-8.71, 7.37) | 0.867 |
| **Oocyte trigger method (%)**   - Dual - hCG - GnRH Agonist | -11.38  -16.83 | (-29.32, 6.56)  (-43.14, 9.46) | 0.206  0.202 |
| **Number of oocytes retrieved (*n*)** | 0.18 | (-0.64, 1.02) | 0.650 |
| **MII oocyte rate (%)** | 0.01 | (-0.51, 0.54) | 0.958 |
| **Fertilization rate (%)** | 0.01 | (-0.43, 0.44) | 0.988 |
| **FF DHEA-S (µg/dL)** | 0.028 | (-0.125, 0.181) | 0.710 |
| **FF Testosterone (ng/dL)** | 0.004 | (-0.008, 0.017) | 0.487 |
| **FF LH (mIU/mL)** | 0.84 | (-1.92, 3.62) | 0.539 |

BMI: Body mass index, PCOM: Polycystic ovarian morphology, AFC: Antral follicle count, GnRH: Gonadotropin-releasing hormone, hCG: Human chorionic gonadotropin, FF: Follicular fluid, DHEA-S: Dehydroepiandrosterone sulfate, LH: Luteinizing hormone; P-values were calculated using univariate linear regression.
