# Supplementary material for: Correlation between follicular fluid hormonal levels in PCOS women and embryo development in ART cycles
Source: PLoS One. 2026 Feb 9;21(2):e0342463. doi: 10.1371/journal.pone.0342463 (PMC12885263; doi:10.1371/journal.pone.0342463)
Supplement: S2 Table — (DOCX) [file pone.0342463.s002.docx]

| Factor | Coefficient | 95% Confident interval (CI) | P-value |
| --- | --- | --- | --- |
| FF Testosterone (ng/mL) | 0.005 | (-0.010, 0.019) | 0.524 |
| FF DHEA-S (ng/mL) | 0.041 | (-0.139, 0.220) | 0.649 |
| FF LH (mIU/mL) | 0.833 | (-2.375, 4.041) | 0.600 |
| Age (years) | 0.384 | (-3.263, 4.031) | 0.831 |
| BMI (kg/m²) | -0.875 | (-2.728, 0.977) | 0.342 |

**S2 Table. Multivariate analysis of factors associated with blastocyst formation rate**

FF: Follicular fluid, DHEA-S: Dehydroepiandrosterone sulfate, LH: Luteinizing hormone, BMI: Body mass index; *P-values were calculated using multivariate linear regression, Mean difference represents the adjusted regression coefficient (β).
