# Supplementary material for: Correlation between follicular fluid hormonal levels in PCOS women and embryo development in ART cycles
Source: PLoS One. 2026 Feb 9;21(2):e0342463. doi: 10.1371/journal.pone.0342463 (PMC12885263; doi:10.1371/journal.pone.0342463)
Supplement: S3 Table — (DOCX) [file pone.0342463.s003.docx]

**S3 Table. Multivariate analysis of factors associated with fertilization rate**

| Factor | Coefficient | 95% Confident interval (CI) | P-value |
| --- | --- | --- | --- |
| FF Testosterone (ng/mL) | -0.011 | (-0.022, -0.001) | 0.040 |
| FF DHEA-S (ng/mL) | -0.105 | (-0.233, 0.023) | 0.105 |
| FF LH (mIU/mL) | 0.900 | (-1.383, 3.183) | 0.427 |
| Age (years) | -2.178 | (-4.774, 0.417) | 0.097 |
| BMI (kg/m²) | 0.101 | (-1.218, 1.419) | 0.877 |

FF: Follicular fluid, DHEA-S: Dehydroepiandrosterone sulfate, LH: Luteinizing hormone, BMI: Body mass index; *P-values were calculated using multivariate linear regression, Mean difference represents the adjusted regression coefficient (β).
